# Exploring and Developing a New Culturally-Appropriate Diabetes Distress Scale in Taiwan

**DOI:** 10.3389/fpubh.2022.838661

**Published:** 2022-03-17

**Authors:** Ching-Ling Lin, Yao-Tsung Chang, Wen-Cheng Liu, Li-Chi Huang, Shin-Yi Tsao, Yu-Hsin Chen, Ruey-Yu Chen

**Affiliations:** ^1^Endocrinology and Metabolism, Cathay General Hospital, Taipei, Taiwan; ^2^School of Medicine, College of Medicine, Taipei Medical University, Taipei, Taiwan; ^3^School of Medicine, National Tsing Hua University, Hsinchu, Taiwan; ^4^School of Public Health, Taipei Medical University, Taipei, Taiwan

**Keywords:** diabetes distress, reliability, validity, factor analysis, scale

## Abstract

**Introduction:**

The aim of this study was to develop and validate a new diabetes distress scale suitable for Chinese and Taiwanese culture.

**Methods:**

This study collected the current diabetes distress measurement tools, re-organized current definitions about the domains of diabetes distress, and then developed a new tool. Three hundred and ninety-five participants from four hospitals in northern Taiwan were recruited by cluster randomized sampling for validity test.

**Results:**

We found the new diabetes distress scale had appropriate reliability and validity, including an acceptable model fit for the 12-item scale.

**Conclusions:**

This new diabetes distress scale might be more directly related to emotional distress issues blood glucose control, improve the clinical conspicuity of diabetes distress, and even benefit the overall care of diabetic patients in Taiwan. Further studies about the validity and reliability of this new tool in a nationwide setting are needed.

## Introduction

Diabetes is a chronic disease that causes comorbidities and mortality and inflicts a heavy burden on medical system and social system worldwide. With the rapid population aging in many countries around the world, the World Health Organization (WHO) notes that the global prevalence rate of diabetes among adults has risen to 8.5% (1). At the same time, WHO also notes that people with diabetes have a 2–3 times higher risk of depression than those without diabetes (2). However, comparing to the diagnosis of depression, “diabetes distress” may be an issue that calls for more attention.

Diabetes distress is defined as emotional burdens, stress, and worries associated with the demanding chronic disease, the blood glucose control or the complications (3). These psychological burdens and worries may further affect patients' mental health and behavior, but they do not reach the level of depression or anxiety. Some studies have found that the worse the blood glucose control, the greater the diabetes distress, which in turn affects self-management behaviors and results in deterioration of blood glucose control (4). Even moderate or severe diabetes distress can seriously interfere with self-management behaviors such as diet and exercise (5), yet, it can be mitigated by improving blood glucose control (6, 7). In Taiwan, the prevalence rate of diabetes among population age 18 and older stands at around 9.82% (8), ranking second place in health insurance outlay (9). Hence, it is particularly important to help patients effectively control their blood glucose to reduce national medical expenditures and improve patients' quality of life. There have been some diabetes distress studies in Taiwan in the past (10, 11); however, since diabetes distress is not routinely evaluated in Taiwan's diabetic share care program, its clinical impact has been overlooked. One of the reasons, we think, may be due to the lack of localized survey tools.

At present, for diabetes distress studies, the most important survey tools used are Problem Areas in Diabetes (PAID) and Diabetes Distress Scale (DDS), which were created by the same author, and DDS was developed based on the PAID to overcome limitations of measures of diabetes distress. In addition to retaining some of the original PAID questions, DDS also integrates additional tools such as the Diabetes Attitude Questionnaire (ATT-39) (12) and Questionnaire on Stress in Patients with Diabetes-Revised (QSD-R).

Currently, PAID-C is the only available tool to measure diabetic distress in Taiwan (13). We decided not to use CDDS, which is the Chinese version of DDS translated by the University of Hong Kong, as we found that most of our patients could not understand the items well-during our pilot study at Cathay General Hospital. It was most likely due to the fact that the difference in idiomatic grammar used in varying parts of Chinese population was not taking into consideration during the translation of DDS (14). At the same time, we also felt that DDS and PAID might lack some items which can sufficiently reflect some important characteristics of local diabetic patients in Taiwan, especially the stress from interpersonal relationships. Although interpersonal relationships such as doctor-patient communication, social support, etc. do affect the blood glucose control and compliance of diabetic patients (15), we have observed that most of our patients seem to attach great importance to “how others view me/my diabetes”—the concept of “face” in traditional Chinese culture (16, 17), resulting in psychological stress, which in turn affects blood glucose control (and health) behavior. Therefore, we decided to design a new diabetes distress scale.

The purpose of this study was to develop and validate Taiwan Diabetes Distress Scale (TDDS) to improve the conspicuity of diabetes distress in Taiwan. In this study, we developed a new diabetes distress measurement tool and conducted a cluster randomized sampling across four division hospitals in our medical center.

## Materials and Methods

Our focus was to develop and validate the TDDS. The study was conducted in three phases to develop a Taiwanese version of diabetes distress scale: (1) reviewing literature, defining domain, and comparing with PAID and DDS (2) item generation, (3) validation test. Data were collected from division hospitals under the Cathay Medical system in Northern Taiwan between September and November 2021. It was approved by the Institutional Review Board (IRB) of Cathay General Hospital.

### Definition of Domains and Design Items

First, we organized an expert group to glean domains and items from PAID and DDS and integrate the experience and opinions of these experts. Then, we redefined or designed new domains and items. The expert group consisted of eight professionals from public health, medicine, and psychology, including two physicians in the department of endocrinology, a psychiatrist, a diabetes health educator, a diabetes health coach, two senior nurses, and a public health professor with extensive experience in scale development. In the first session, we integrated the definition and framework from PAID and DDS (18), collected PAID-C and CDDS items from literature (10, 13, 19), and designed some new items based on our past subjective experience in educating and coaching diabetic patients. We took reference of Schmitt et al. (18) published article, which compares PAID and DDS item by item, to devise a complete questionnaire item. It allowed us to easily measure and compare the applicability of each item and domain with the local culture in Taiwan and determine whether to modify the original item or add some new items.

After careful evaluation, we listed 5 diabetes distress domains: (1) Diabetes complications, (2) Diabetes self-management, (3) Treatment-related problems, (4) Social and environment support, and (5) Emotional burden. Table 1 contains a list of our domain definitions. In addition, the expert group believed the diabetes distress scale should focus on patients' subjective feelings, attitudes, and emotion. Therefore, we generated a total of 31 items to cover these domains, and every item began with the heading “I am worried about…,” “Because of…, it makes me feel stressed,” or “When I …, it makes me feel anxiety”. In addition, as mentioned, based on the long-term interactive experience of the expert team in treating or counseling patients, we believed that stress from interpersonal relationships especially the concern of “face” was indeed one of the factors that affect diabetes distress. For example, sentiments like the fear of being blamed by doctors for poor blood glucose control, incomprehension of diabetes health education but dare not to raise questions, or disinclination to become a burden to children prompted us to design some questions that adequately expressed “I care about what others think of me, and it creates pressure on me” based on common patients' responses. Then we checked the feasibility and content validity based on the five-point Likert Scale and open-ended feedback. Items remaining on the list were dependent on the four criteria: (1) fitness ≧ 3.0 points, (2) importance ≧ 3.0 points, (3) description clarity ≧ 3.0 points, and (4) Evaluators' specific amendments. In addition to our expert group, 15 additional healthcare workers with diabetes from Cathay General Hospital were recruited to conduct a simple pilot study to further revise the sentence in each item.

**Table 1:** Definitions of the Taiwanese diabetes distress scale.

**Domains**	**Definition**

Feel worry, fear of, or have stress about…	
Diabetes complications	Worry about diabetes complications in the next few years or sudden hypoglycemia events
Diabetes self-management	Worry about deterioration of blood glucose control due to lifestyle issues (e.g., diet, exercise, and substance use)
Treatment-related problems	Worry about not being able to understand the content of treatment and health education or communicate with doctors
Social and environment support	Feeling stressed about difficulty in controlling blood glucose due to insufficient social support or unsupported living environment and working conditions
Emotional burden	Emotional burden on diabetes and self-management behavior

Finally, the first version of TDDS contained 31 questions with descriptions of some items being corrected. Different from PAID and DDS, TDDS adopted tools such as the catastrophe scale with a scale of 0-10 points (20) to provide a visual score scale which could effectively reflect the state of stress. On the questionnaire, 0 denoted the least worried or least stressful while 10 being extremely worried or extremely stressful. The diabetes complications consisted of seven items, including the worry about poor blood glucose control/dialysis/eye disease/increasing dosage/foot disease/cardiovascular disease/hypoglycemic events; the diabetes self-management consisted of six items, including the worry about poor blood glucose control due to poor eating habits/insufficient exercise/forgetting to take medicine or insulin/poor self-monitoring of blood glucose/smoking/drinking; treatment-related problems consisted of six items, including the worry about being blamed by the doctor/asking the doctor or the health educator questions/feeling sorry for not meeting doctor's expectations; the social and environment support consisted of six items, including the stress from insufficient family support/impact of job requirements/relationship pressure/limitation of living environment; and finally the emotional burden consisted of six items, including the anxiety of regular hospital visits/diabetes/the need of lifestyle changes due to diabetes/being known to others as of having diabetes. Considering the original survey items were constructed in Chinese language, for more detailed information about specific items, please contact the author(s).

### Samples and Sample Size

Due to the difference in regions, types of hospitals, and patients' characteristics, we adopted the cluster randomized sampling method and allocated the sampling proportion according to the number of patients with type 2 diabetes in the four hospitals of Cathay Medical system. According to the recommendations of sample size for conducting factor analysis (21), the sample size we decided to use was 10 times the number of questions for factor analysis. In addition, considering that most of our patients were elderly, possibly having a lower education level and unable to provide complete answers, we had set a higher number of invalid samples to ensure enough valid samples. Therefore, with the intention to distribute at least 310 questionnaires plus about 20% of invalid samples, we finally came up with a total of 400 questionnaires to be distributed. The Cathay Medical system currently has four hospitals in Taiwan, namely a medical center, two regional hospitals, and a clinic, with three hospitals located in Taipei and the fourth one in Hsinchu. According to the proportion of total number of patients with type 2 diabetes in each hospital, we allocated 170, 100, 80, and 50 questionnaires to the Cathay General Hospital, Sijhih Branch, Hsinchu Branch, and Neihu Clinic.

### Statistical Analysis

The validation test included exploratory factor analysis and confirmatory factor analysis. The exploratory factor analysis was used for reducing the number of items. We used the principal component analysis with the varimax rotation and eigenvalue criterion > 1.0 to detect the latent variable. Items with factor loading <0.50, cross-loading > 0.40, or communalities <0.30 were eliminated (22, 23), and every latent variable had to have at least three factors. It was analyzed at a 95% significance level and conducted using PASW 22.0 software for windows (SPSS, Chicago, IL).

Then, we performed the confirmatory factor analysis to build the conceptual model and compare it with the original model as Table 1 to establish construct validity and reliability of TDDS. We tested the model fit for every latent variable separately before building a full model to detect any unsuitable item. The following criteria of model fit were used: root mean square error of approximation (RMSEA) <0.08, χ^2^/df <5, standardized root mean square residual (SRMR) <0.08, comparative fit index (CFI) > 0.90, Tucker-Lewis index (TLI) > 0.90 (24–27). In addition, the composite reliability (CR) needed to be >0.7 for appropriate construct reliability, and average variance extracted (AVE) had to be >0.5 for appropriate convergent validity (28, 29). We used AMOS 20.0 software to conduct confirmatory factor analysis (Chicago, IL).

Finally, we tested the Cronbach's α for appropriate content reliability (30), and the threshold was 0.70 or greater. In addition, we used a chi-square test or an ANOVA test to test the differences in demographic characteristics of participants between the four hospitals. We also assessed participants' mental health by using the five-item Brief Symptom Rating Scale (BSRS-5), which has good reliability and validity (31). It is a five-item, self-rated questionnaire with each item ranging from 0 (not at all) to 4 (extremely). We used Pearson correlation coefficient to initially analyze the correlation between HbA1c, mental health, and the final questionnaire score in an attempt to prove that our new scale could take into account the sensitivity of PAID and DDS to blood glucose and mental health.

## Results

### Demographics

Table 2 shows the demographic characteristics of the 395 valid samples: 54.7% were males, the mean age was 57.5 years (SD = 12.68), 48.2% had a bachelor's degree or higher, the mean HbA1c was 7.19% (SD = 1.30), average duration of diabetes treatment was 10.22 years (SD = 8.23), and about 12.9% participants had medium or high pressure. There are significant differences in age, education level and duration of diabetes treatment of the participants from the four hospitals.

**Table 2:** Demographic characteristics of samples.

	**Cathay Hospital Systems**, ***N*** **(%)**				***p*-value**
	**General Hospital (*n* = 164)**	**Sijhih Branch (*n* = 100)**	**Neihu Clinic (*n* = 51)**	**Hsinchu Branch (*n* = 80)**	
**Gender**					0.055
Male	86 (52.4)	48 (48.0)	36 (70.6)	46 (57.5)	
Female	78 (47.6)	52 (52.0)	15 (29.4)	34 (42.5)	
**Age, years**					0.001**
18–39	15 (9.3)	17 (17.0)	0 (0.0)	5 (6.2)	
40–49	20 (12.3)	11 (11.0)	14 (27.5)	19 (23.8)	
50–64	72 (44.4)	47 (47.0)	23 (45.1)	25 (31.2)	
65 or higher	55 (34.0)	25 (25.0)	14 (27.5)	31 (38.8)	
Age, mean ± SD	59.41 ± 13.12	54.43 ± 13.07	57.02 ± 9.79	57.91 ± 12.37	0.021*
**Education level**					0.004**
Junior high school and bellow	29 (17.8)	28 (28.0)	4 (7.8)	25 (31.2)	
Senior high school	46 (28.2)	35 (35.0)	13 (25.5)	24 (30.0)	
University or professional study	70 (42.9)	32 (32.0)	30 (58.8)	28 (35.0)	
Master's degree or above	18 (11.0)	5 (5.0)	4 (7.8)	3 (3.8)	
Duration of diabetes treatment (years, mean ± SD)	10.23 ± 8.55	8.28 ± 7.15	9.62 ± 6.46	13.06 ± 9.12	0.002**
HbA1c (%, mean ± SD)	7.01 ± 1.23	7.42 ± 1.69	7.38 ± 1.06	7.14 ± 0.90	0.067
**Mental Health**					0.153
Normal	119 (72.6)	63 (63.0)	32 (62.7)	51 (63.8)	
Light pressure	30 (18.3)	17 (17.0)	12 (23.5)	20 (25.0)	
Medium pressure	10 (6.1)	14 (14.0)	6 (11.8)	9 (11.2)	
High pressure	5 (3.0)	6 (6.0)	1 (2.0)	0 (0.0)	
Mental Health, mean ± SD	3.75 ± 3.87	5.23 ± 4.89	4.39 ± 4.04	4.34 ± 3.92	0.052

*0.01 < p < 0.05, **p < 0.01.

### Exploratory Factor Analysis

The first exploratory factor analysis produced four latent variables, accounting for 72.05% of the total variance. The KMO test of sampling adequacy was 0.957, and the Bartlett's test for sphericity was highly significant (*p* < 0.001). Then, the original 31 items were reviewed for possible elimination according to their factor loading and cross-loading criteria. There were eight items eliminated, which made the latent variables reduced to three, and the total variation explanatory power was reduced to 69.93%. The social and environment support and the emotional burden domains were classified into the same domain under factor analysis, a total of 12 questions accounting for 55.26% of the total variance, and the diabetes self-management domain was completely excluded. Therefore, we changed the combined domain's label to “Life and interpersonal stress”. In addition, since the health education related items were excluded from the treatment-related problems domain, only four items about the problem of communicating with doctors were left, so we changed the label to the problem of “communication concerns”. The fear of diabetes complications domain had five items remaining.

### Confirmatory Factor Analysis

The confirmatory factor analysis tested each of the three domains after conducting the exploratory factor analysis. The first domain–“Life and interpersonal stress”–had 12 items, and we eliminated Q20, Q22, Q23, Q27, Q29, and Q30 since they had higher modification index (M.I.) value and high significant correlation with other items, and this made the final RMSEA = 0.083, χ^2^/df = 3.698, and CFI and TLI were both over 0.98. Although removing Q26 could reduce RMSEA to 0.045 and χ^2^/df to 1.815, we still kept this until the full model analysis for its importance. The second domain–“Fear of diabetes complications”–had five items. We eliminated Q7 for the same reason, and this made the final RMSEA = 0.089, χ^2^/df = 4.092, and CFI and TLI were both over 0.98. The third domain–“Communication concerns”–had four items, and we eliminated Q17 since it had significant correlation with other three items.

Finally, in the full model analysis, we eliminated Q6 due to its high M.I. value and made the final RMSEA = 0.079, χ^2^/df = 3.464, and CFI and TLI were both over 0.95. If we removed Q26, RMSEA would only drop to 0.077, and χ^2^/df would drop to 3.308, which did not show much improvement, so we finally decided to keep Q26 (Table 3). All the domains could meet the criteria of CR and AVE, and the Cronbach's α was 0.863–0.924. The item-total correlation of each item was 0.685–0.799, and the correlation between subscales was 0.577–0.660. The final model had 12 items in total, accounting for 75.27% of the total variance. In addition, the total points of final model were significantly related to the HbA1c (Pearson correlation = 0.304, *p* < 0.001) and the mental health (Pearson correlation = 0.452, *p* < 0.001).

**Table 3:** Factor loading for 12-items diabetes distress scale (*N* = 390).

**Domain**	**Original item code**	**Cronbach's α**	**Corrected item-to-total correlation**	**Factor loadings**			**CR**	**AVE**	**Total explained variance (%)**
				**1**	**2**	**3**			
Life and interpersonal stress	Q21	0.924	0.799	0.659			0.896	0.590	55.28
	Q24		0.697	0.766					
	Q25		0.758	0.710					
	Q26		0.777	0.793					
	Q28		0.794	0.712					
	Q31		0.685	0.750					
Fear of Diabetes complications	Q3	0.863	0.719		0.844		0.928	0.812	11.45
	Q4		0.739		0.896				
	Q5		0.733		0.875				
Communication Concerns	Q14	0.896	0.745			0.868	0.871	0.694	8.54
	Q15		0.740			0.840			
	Q16		0.732			0.684			

*CR, composite reliability; AVE, average variance extracted*.

## Discussion

In this study, we developed the first Diabetes Distress Scale that was specifically based on Chinese culture, and it had acceptable construct validity, content validity, composite reliability, and internal consistency. This Chinese version of the scale was developed based on the two previously translated and verified diabetes distress questionnaires, and it was compiled and modified based on the unique local culture in Taiwan which we learned from our daily clinical treatment, coaching, and health education experience. We believe it can pertinently reflect the current status of patients with type 2 diabetes in Taiwan and can be used to enhance diabetes distress prevention and improve diabetes treatment.

Comparing to PAID, PAID-C, DDS and CDDS, TDDS uses more consistent sentences to describe feelings of worry and anxiety. For example, PAID mostly starts with “Feeling” or “Worrying,” and covers a variety of negative emotions such as anger, overwhelm, concern, unsatisfaction, and depression. However, the consistency of the sentence is no longer present after the original sentence is translated into Chinese in PAID-C, and it may make PAID-C less sensitive to the detection of diabetes distress, causing it to have less correlation with changes in blood glucose control (11). On the contrary, although DDS also starts with “feeling,” the content is mostly related to dissatisfaction or disappointment about something, such as feeling unsupported, feeling unconfident, feeling not working hard enough to control blood glucose, etc. These items were not directly related to emotional reactions as PAID, so the connection with depression is reduced (18). Therefore, with highly consistent emotional narratives, we believe that not only can TDDS be more sensitive to emotional distress, but it also takes into account the relevance of blood glucose control, and the analysis indeed exhibits that the score of TDDS is significantly related to HbA1c and psychology.

Another notable feature of this study is the consideration of the impact of the concept of “face” in traditional Chinese culture on the emotional distress of diabetic patients in Taiwan. As opposed to the individual-oriented approach toward social interaction in Western countries, individuals in Eastern countries customarily have a social-oriented approach toward their communities (17, 32, 33). This makes the Chinese more concerned about “how others think of me” than Westerners and possibly bothered by it. That is, the concern of “face” is one of the most important concepts in individuals' decisions concerning the relationship with others in Chinese culture (17), and its influence may even exceed the focus on one's own health behaviors, making the concerns about diet, exercise, blood glucose measurement and other behaviors that were originally emphasized in PAID and DDS become relatively less important in TDDS. As for issues in mental health and distress problems, this unique culture causes Chinese people to be more stressed when facing various health related issues because they are concerned about how others think of themselves and may even cause them to ignore their own responsibilities for their behavior choice.

TDDS's direct connection with the feeling of worry and anxiety also enables its users to directly coach on specific emotions. From the perspective of coaching and psychological counseling, emotional distress may come from irrational beliefs, in other words, thoughts that are out of touch with facts (34, 35). Irrational beliefs and emotions may prevent patients from changing actual behaviors, so even if actual behaviors and blood glucose control results are getting some improvement, they may not be effective in alleviating emotional distress. This may be one of the reasons why many studies found that blood glucose control is not significantly related to changes in PAID or DDS scores, Compared with PAID and DDS, which are mostly negative feelings for specific events or phenomena, TDDS might better capture the emotional distress caused by irrational beliefs so that appropriate methods can be introduced to help remove irrational emotions as we strongly believe that improving diabetes distress may improve blood glucose control (36–38).

Hence, there are several strengths in this study. First, we conducted a complete review of the current diabetes distress measurement tools and literature. This allowed us to specifically review the advantages and disadvantages of existing tools and redesign a new scale based on the psychological and cultural characteristics of the Chinese, thereby increasing the applicability and reliability of the TDDS scale in the Chinese environment. Second, we sampled at different levels of hospitals, both urban and rural areas, to ensure the diversity of the samples and deliver a representative study. Third, we examined diabetes distress problems from the perspective of professional psychological counseling and designed consistent questions so that users of this scale will be able to coach patients more effectively to improve emotional distress problems, hence making this scale realistically and accurately measure diabetes distress. Finally, the final version of TDDS has only 12 items, which is fewer than the original PAID and DDS, and has good reliability and validity as these two. Its convenience in clinical use not only improves the efficiency and accuracy of clinical screening for diabetes distress but also facilitates/improves diabetes distress treatment or coaching.

The limitations of this study could be used to build future research. First, an effective, validated representation of TDDS should be tested in more countries to verify whether the Chinese in different regions do have similar cultural characteristics, and whether TDDS is suitable for translating into other languages. Second, we would take different weights to each domain of TDDS into consideration in the future since it has an unbalanced number of items in each domain. Therefore, for future studies, we suggest that a longitudinal study should be designed to test whether TDDS is indeed sufficiently sensitive to identify and help/aid in coaching on diabetes distress, and whether it can be applied to Chinese in other countries.

## Conclusion

In this study, we developed and validated TDDS, and the results of this study indicated TDDS has appropriate reliability and validity. TDDS is suitable for measuring the worry and anxiety about diabetes complications, taking into account the communication issue, and the concern of “face” in patients with type 2 diabetes. Based on the limitations and strengths of current study, we suggest that longitudinal studies need to be done for the validity and reliability of TDDS in the management of diabetes distress in a nationwide setting.

## Data Availability Statement

The raw data supporting the conclusions of this article will be made available by the authors, without undue reservation.

## Ethics Statement

The studies involving human participants were reviewed and approved by Institutional Review Board of Cathay General Hospital (Ref no. CGH-OP110039). Written informed consent for participation was not required for this study in accordance with the national legislation and the institutional requirements.

## Author Contributions

C-LL, Y-TC, and R-YC organized and conducted the study, designed the questionnaire, interpreted the results, proposed the structure of the paper, and formulated the paper. Y-TC and R-YC performed the statistical analysis. C-LL and R-YC managed the research project. C-LL, W-CL, L-CH, S-YT, and Y-HC collected data from their patients, critically appraised the paper, and made final suggestions. C-LL, Y-TC, and R-YC are the guarantors of this work and had full access to all the data in the study and take responsibility for the integrity of the data and the accuracy of the data analysis. All authors reviewed and revised the manuscript and agreed to the submission of the final manuscript.

## Conflict of Interest

The authors declare that the research was conducted in the absence of any commercial or financial relationships that could be construed as a potential conflict of interest.

## Publisher's Note

All claims expressed in this article are solely those of the authors and do not necessarily represent those of their affiliated organizations, or those of the publisher, the editors and the reviewers. Any product that may be evaluated in this article, or claim that may be made by its manufacturer, is not guaranteed or endorsed by the publisher.

## Abbreviations

TDDS: Taiwan Diabetes Distress Scale
PAID: Problem Areas in Diabetes
DDS: Diabetes Distress Scale
WHO: World Health Organization
QSD-R: Questionnaire on Stress in Patients with Diabetes-Revised
RMSEA: root mean square error of approximation
SRMR: standardized root mean square residual
CFI: comparative fit index
TLI: Tucker-Lewis index
CR: composite reliability
AVE: average variance extracted.
